# Supplementary material for: Hot spot prediction in protein-protein interactions by an ensemble system
Source: BMC Syst Biol. 2018 Dec 31;12(Suppl 9):132. doi: 10.1186/s12918-018-0665-8 (PMC6311905; doi:10.1186/s12918-018-0665-8)
Supplement: Supplementary file 2 — All relevant MATLAB code. A simple Matlab implement of our predictor. (ZIP 19,742 kb) [file 12918_2018_665_MOESM2_ESM.zip › Additional file 2/Read ASA/ASA website/dbMPIKT/4OZGE.html]

 NetSurfP - Protein Surface Accessibility and Secondary Structure Predictions

  

|  |  |  |
| --- | --- | --- |
|  |  | NetSurfP - Protein Surface Accessibility and Secondary Structure Predictions  Technical University of Denmark |

  


---

```
# For publication of results, please cite:
# A generic method for assignment of reliability scores applied to solvent accessibility predictions.
# Bent Petersen, Thomas Nordahl Petersen, Pernille Andersen, Morten Nielsen and Claus Lundegaard
# BMC Structural Biology 2009, 9:51 doi:10.1186/1472-6807-9-51
#
# Column 1: Class assignment - B for buried or E for Exposed - Threshold: 25% exposure, but not based on RSA
# Column 2: Amino acid
# Column 3: Sequence name
# Column 4: Amino acid number
# Column 5: Relative Surface Accessibility - RSA
# Column 6: Absolute Surface Accessibility
# Column 7: Z-fit score for RSA prediction
# Column 8: Probability for Alpha-Helix
# Column 9: Probability for Beta-strand
# Column 10: Probability for Coil
E M  4OZGE                  1    0.523 104.572  -1.445   0.005   0.045   0.951
E K  4OZGE                  2    0.469  96.494   0.842   0.004   0.616   0.381
B T  4OZGE                  3    0.285  39.529  -0.830   0.004   0.616   0.381
E T  4OZGE                  4    0.381  52.817  -0.371   0.004   0.420   0.576
E Q  4OZGE                  5    0.412  73.601  -0.272   0.004   0.138   0.858
E P  4OZGE                  6    0.437  62.067  -0.333   0.018   0.047   0.935
E P  4OZGE                  7    0.573  81.337  -0.998   0.053   0.043   0.903
E S  4OZGE                  8    0.406  47.536  -1.212   0.066   0.296   0.638
B M  4OZGE                  9    0.212  42.501  -0.024   0.023   0.655   0.322
E D  4OZGE                 10    0.468  67.511  -0.468   0.021   0.756   0.223
B C  4OZGE                 11    0.251  35.184   0.162   0.022   0.552   0.426
E A  4OZGE                 12    0.433  47.695   0.578   0.021   0.279   0.699
E E  4OZGE                 13    0.537  93.796   0.404   0.004   0.085   0.910
E G  4OZGE                 14    0.636  50.061  -1.079   0.005   0.045   0.951
E R  4OZGE                 15    0.424  97.073   0.407   0.004   0.197   0.799
E A  4OZGE                 16    0.552  60.797   1.110   0.003   0.718   0.279
B A  4OZGE                 17    0.073   8.078  -0.060   0.001   0.959   0.040
E N  4OZGE                 18    0.423  61.898   1.238   0.001   0.959   0.040
B L  4OZGE                 19    0.053   9.778   0.254   0.001   0.959   0.040
E P  4OZGE                 20    0.394  55.909   1.132   0.001   0.900   0.099
B C  4OZGE                 21    0.029   4.100   0.109   0.001   0.900   0.099
E N  4OZGE                 22    0.420  61.561   0.908   0.001   0.900   0.099
B H  4OZGE                 23    0.224  40.691  -0.805   0.002   0.816   0.182
B S  4OZGE                 24    0.348  40.809  -0.889   0.003   0.718   0.279
E T  4OZGE                 25    0.491  68.143  -2.037   0.005   0.336   0.660
E I  4OZGE                 26    0.378  69.874  -2.371   0.004   0.138   0.858
E S  4OZGE                 27    0.430  50.373  -1.424   0.018   0.088   0.893
E G  4OZGE                 28    0.512  40.334  -2.154   0.018   0.088   0.893
E N  4OZGE                 29    0.340  49.747  -1.244   0.004   0.138   0.858
B E  4OZGE                 30    0.247  43.133  -0.406   0.005   0.336   0.660
B Y  4OZGE                 31    0.097  20.622   0.309   0.003   0.718   0.279
B V  4OZGE                 32    0.026   3.919   0.672   0.018   0.846   0.136
B Y  4OZGE                 33    0.055  11.818   0.464   0.011   0.918   0.071
B W  4OZGE                 34    0.024   5.868   0.821   0.011   0.918   0.071
B Y  4OZGE                 35    0.035   7.565   0.469   0.011   0.918   0.071
B R  4OZGE                 36    0.122  28.030   0.612   0.018   0.846   0.136
B Q  4OZGE                 37    0.082  14.717   0.717   0.023   0.655   0.322
E I  4OZGE                 38    0.344  63.603   1.754   0.004   0.085   0.910
E H  4OZGE                 39    0.402  73.033   0.388   0.005   0.015   0.979
E S  4OZGE                 40    0.845  99.057  -1.679   0.003   0.003   0.994
E Q  4OZGE                 41    0.405  72.333   0.257   0.005   0.015   0.979
E G  4OZGE                 42    0.397  31.220   0.093   0.018   0.088   0.893
B P  4OZGE                 43    0.080  11.309   0.358   0.005   0.336   0.660
B Q  4OZGE                 44    0.233  41.650   0.541   0.002   0.816   0.182
B Y  4OZGE                 45    0.091  19.340   0.533   0.001   0.959   0.040
B I  4OZGE                 46    0.023   4.218   1.138   0.001   0.959   0.040
B I  4OZGE                 47    0.022   4.033   0.714   0.001   0.959   0.040
B H  4OZGE                 48    0.090  16.444   0.323   0.000   0.983   0.017
B G  4OZGE                 49    0.099   7.799  -0.122   0.001   0.959   0.040
B L  4OZGE                 50    0.175  32.134  -0.813   0.003   0.718   0.279
B K  4OZGE                 51    0.261  53.791  -1.005   0.004   0.420   0.576
E N  4OZGE                 52    0.622  91.090  -1.886   0.004   0.085   0.910
E N  4OZGE                 53    0.467  68.310  -1.045   0.005   0.045   0.951
E E  4OZGE                 54    0.555  96.958  -1.025   0.018   0.088   0.893
E T  4OZGE                 55    0.708  98.172  -1.026   0.018   0.088   0.893
E N  4OZGE                 56    0.609  89.187  -1.582   0.004   0.138   0.858
E E  4OZGE                 57    0.367  64.097  -0.876   0.004   0.138   0.858
E M  4OZGE                 58    0.426  85.323  -0.838   0.005   0.336   0.660
B A  4OZGE                 59    0.180  19.803  -2.781   0.004   0.616   0.381
B S  4OZGE                 60    0.186  21.787  -0.622   0.002   0.816   0.182
B L  4OZGE                 61    0.043   7.818  -1.025   0.001   0.959   0.040
B I  4OZGE                 62    0.206  38.128   0.110   0.001   0.959   0.040
B I  4OZGE                 63    0.063  11.655  -1.468   0.001   0.900   0.099
E T  4OZGE                 64    0.300  41.555  -0.212   0.002   0.816   0.182
E E  4OZGE                 65    0.350  61.180  -0.528   0.004   0.514   0.481
E D  4OZGE                 66    0.585  84.371  -1.345   0.004   0.138   0.858
E R  4OZGE                 67    0.655 149.881  -1.159   0.005   0.015   0.979
E K  4OZGE                 68    0.469  96.432  -0.400   0.004   0.197   0.799
E S  4OZGE                 69    0.365  42.813   0.527   0.003   0.718   0.279
B S  4OZGE                 70    0.056   6.587   0.011   0.001   0.900   0.099
B T  4OZGE                 71    0.191  26.464   0.398   0.001   0.900   0.099
B L  4OZGE                 72    0.023   4.211   0.629   0.001   0.959   0.040
B I  4OZGE                 73    0.240  44.382   0.525   0.001   0.959   0.040
B L  4OZGE                 74    0.073  13.458  -0.138   0.001   0.900   0.099
E P  4OZGE                 75    0.459  65.075   0.145   0.004   0.616   0.381
E H  4OZGE                 76    0.461  83.783  -0.648   0.005   0.336   0.660
B A  4OZGE                 77    0.092  10.127   0.005   0.004   0.197   0.799
E T  4OZGE                 78    0.428  59.391   0.785   0.004   0.197   0.799
E L  4OZGE                 79    0.349  63.957  -0.595   0.019   0.141   0.840
E R  4OZGE                 80    0.505 115.737  -0.864   0.018   0.047   0.935
B D  4OZGE                 81    0.209  30.131  -0.975   0.018   0.088   0.893
B T  4OZGE                 82    0.199  27.615  -0.042   0.022   0.359   0.619
B A  4OZGE                 83    0.057   6.325  -0.753   0.003   0.718   0.279
B V  4OZGE                 84    0.120  18.429   0.482   0.001   0.900   0.099
B Y  4OZGE                 85    0.032   6.796   0.774   0.001   0.900   0.099
B Y  4OZGE                 86    0.048  10.172   0.646   0.001   0.900   0.099
B C  4OZGE                 87    0.013   1.867   1.416   0.001   0.900   0.099
B I  4OZGE                 88    0.038   6.956  -0.182   0.002   0.816   0.182
B V  4OZGE                 89    0.063   9.698  -0.553   0.003   0.718   0.279
B L  4OZGE                 90    0.124  22.741  -0.285   0.004   0.514   0.481
E G  4OZGE                 91    0.344  27.073  -1.465   0.005   0.262   0.733
E G  4OZGE                 92    0.375  29.505  -2.708   0.004   0.085   0.910
B A  4OZGE                 93    0.295  32.454  -1.680   0.018   0.088   0.893
E D  4OZGE                 94    0.369  53.115  -1.560   0.018   0.047   0.935
B G  4OZGE                 95    0.264  20.745  -1.052   0.019   0.141   0.840
B L  4OZGE                 96    0.229  41.893  -0.551   0.021   0.279   0.699
B T  4OZGE                 97    0.091  12.580  -2.124   0.021   0.451   0.528
B F  4OZGE                 98    0.079  15.835  -2.064   0.021   0.451   0.528
B G  4OZGE                 99    0.078   6.162  -4.097   0.005   0.336   0.660
E K  4OZGE                100    0.328  67.387  -0.674   0.004   0.197   0.799
B G  4OZGE                101    0.240  18.872  -1.660   0.004   0.085   0.910
E T  4OZGE                102    0.325  45.064  -1.106   0.005   0.262   0.733
E H  4OZGE                103    0.466  84.711  -0.235   0.004   0.616   0.381
B L  4OZGE                104    0.105  19.134  -0.156   0.003   0.718   0.279
E I  4OZGE                105    0.306  56.536   0.042   0.018   0.846   0.136
B I  4OZGE                106    0.168  31.043  -0.314   0.011   0.918   0.071
E Q  4OZGE                107    0.384  68.529   0.057   0.018   0.846   0.136
E P  4OZGE                108    0.412  58.420  -1.237   0.023   0.655   0.322
E Y  4OZGE                109    0.532 113.582  -0.522   0.021   0.279   0.699
E I  4OZGE                110    0.461  85.230  -1.535   0.019   0.141   0.840
E Q  4OZGE                111    0.617 110.250  -0.932   0.019   0.141   0.840
E N  4OZGE                112    0.478  69.979  -0.993   0.005   0.045   0.951
E P  4OZGE                113    0.323  45.862  -0.772   0.005   0.045   0.951
E D  4OZGE                114    0.588  84.702  -0.369   0.005   0.045   0.951
B P  4OZGE                115    0.180  25.528  -1.265   0.004   0.138   0.858
E A  4OZGE                116    0.284  31.341  -1.171   0.004   0.420   0.576
B V  4OZGE                117    0.226  34.782  -0.945   0.003   0.718   0.279
B Y  4OZGE                118    0.200  42.804  -0.703   0.023   0.655   0.322
E Q  4OZGE                119    0.341  60.903   0.006   0.021   0.451   0.528
B L  4OZGE                120    0.091  16.735  -2.055   0.021   0.279   0.699
E R  4OZGE                121    0.425  97.279  -0.163   0.004   0.197   0.799
E D  4OZGE                122    0.611  88.016  -0.921   0.004   0.138   0.858
E S  4OZGE                123    0.513  60.112  -1.319   0.018   0.047   0.935
E K  4OZGE                124    0.748 153.946  -2.034   0.018   0.019   0.964
E S  4OZGE                125    0.533  62.479  -1.925   0.018   0.019   0.964
E S  4OZGE                126    0.523  61.307   0.238   0.005   0.045   0.951
B D  4OZGE                127    0.222  32.005   0.190   0.005   0.336   0.660
B K  4OZGE                128    0.168  34.578   0.306   0.002   0.816   0.182
B S  4OZGE                129    0.059   6.903   0.174   0.001   0.900   0.099
B V  4OZGE                130    0.029   4.442   0.663   0.001   0.900   0.099
B C  4OZGE                131    0.014   1.980   1.633   0.001   0.900   0.099
B L  4OZGE                132    0.034   6.152   0.309   0.001   0.900   0.099
B F  4OZGE                133    0.031   6.161   0.047   0.003   0.718   0.279
B T  4OZGE                134    0.166  23.080   0.051   0.005   0.336   0.660
E D  4OZGE                135    0.397  57.179  -1.320   0.004   0.197   0.799
B F  4OZGE                136    0.120  24.144  -0.330   0.004   0.085   0.910
B D  4OZGE                137    0.226  32.581   0.296   0.005   0.045   0.951
B S  4OZGE                138    0.239  28.058  -1.140   0.018   0.047   0.935
E Q  4OZGE                139    0.529  94.551  -0.684   0.053   0.043   0.903
B T  4OZGE                140    0.240  33.330  -0.969   0.052   0.084   0.864
B N  4OZGE                141    0.199  29.104  -0.545   0.022   0.359   0.619
B V  4OZGE                142    0.209  32.077  -0.602   0.021   0.756   0.223
B S  4OZGE                143    0.224  26.229  -0.193   0.018   0.846   0.136
B Q  4OZGE                144    0.207  36.899  -0.157   0.011   0.918   0.071
B S  4OZGE                145    0.274  32.136  -0.053   0.018   0.846   0.136
E K  4OZGE                146    0.403  82.835   0.027   0.023   0.655   0.322
E D  4OZGE                147    0.618  89.111  -1.263   0.021   0.279   0.699
E S  4OZGE                148    0.661  77.504  -2.293   0.005   0.045   0.951
E D  4OZGE                149    0.622  89.616  -1.479   0.005   0.262   0.733
E V  4OZGE                150    0.519  79.801  -1.253   0.004   0.514   0.481
E Y  4OZGE                151    0.370  78.984  -1.244   0.004   0.514   0.481
E I  4OZGE                152    0.448  82.899  -1.149   0.004   0.420   0.576
E T  4OZGE                153    0.502  69.627  -1.580   0.005   0.262   0.733
E D  4OZGE                154    0.481  69.341  -1.569   0.004   0.138   0.858
E K  4OZGE                155    0.525 108.034  -0.440   0.005   0.262   0.733
E C  4OZGE                156    0.361  50.642  -1.399   0.004   0.616   0.381
E V  4OZGE                157    0.359  55.101  -0.365   0.002   0.816   0.182
E L  4OZGE                158    0.320  58.629   0.152   0.001   0.900   0.099
E D  4OZGE                159    0.480  69.110   0.513   0.002   0.816   0.182
E M  4OZGE                160    0.287  57.329  -0.118   0.003   0.718   0.279
E R  4OZGE                161    0.574 131.400  -0.558   0.004   0.420   0.576
E S  4OZGE                162    0.630  73.789  -1.887   0.004   0.197   0.799
E M  4OZGE                163    0.539 107.894  -1.661   0.004   0.138   0.858
E D  4OZGE                164    0.586  84.443  -2.072   0.005   0.262   0.733
B F  4OZGE                165    0.213  42.669  -1.874   0.004   0.616   0.381
B K  4OZGE                166    0.177  36.327  -0.592   0.002   0.816   0.182
B S  4OZGE                167    0.111  13.009  -0.419   0.001   0.900   0.099
B N  4OZGE                168    0.068   9.941   0.023   0.001   0.900   0.099
B S  4OZGE                169    0.073   8.556  -0.432   0.001   0.900   0.099
B A  4OZGE                170    0.050   5.543  -0.856   0.011   0.918   0.071
B V  4OZGE                171    0.093  14.325  -1.060   0.011   0.918   0.071
B A  4OZGE                172    0.189  20.850  -0.518   0.001   0.900   0.099
B W  4OZGE                173    0.123  29.461  -0.992   0.004   0.616   0.381
E S  4OZGE                174    0.474  55.588   0.353   0.004   0.138   0.858
E N  4OZGE                175    0.630  92.276   0.369   0.005   0.015   0.979
E K  4OZGE                176    0.764 157.155  -0.504   0.005   0.015   0.979
E S  4OZGE                177    0.621  72.758   0.244   0.004   0.138   0.858
E D  4OZGE                178    0.555  79.961   1.451   0.003   0.718   0.279
B F  4OZGE                179    0.093  18.585   1.247   0.001   0.900   0.099
E A  4OZGE                180    0.444  48.907   1.716   0.001   0.900   0.099
B C  4OZGE                181    0.031   4.395  -0.880   0.001   0.900   0.099
E A  4OZGE                182    0.411  45.325   1.608   0.011   0.918   0.071
B N  4OZGE                183    0.106  15.504  -0.314   0.011   0.918   0.071
E A  4OZGE                184    0.514  56.621   0.958   0.001   0.900   0.099
B F  4OZGE                185    0.189  37.993   0.037   0.003   0.718   0.279
E N  4OZGE                186    0.702 102.846   0.558   0.004   0.085   0.910
E N  4OZGE                187    0.686 100.386  -0.953   0.005   0.015   0.979
E S  4OZGE                188    0.635  74.457  -0.290   0.005   0.336   0.660
E I  4OZGE                189    0.473  87.505  -0.182   0.003   0.718   0.279
E I  4OZGE                190    0.463  85.692   0.734   0.021   0.756   0.223
E P  4OZGE                191    0.366  51.907   0.262   0.023   0.655   0.322
E E  4OZGE                192    0.448  78.318  -0.877   0.021   0.451   0.528
E D  4OZGE                193    0.545  78.462  -0.235   0.021   0.279   0.699
E T  4OZGE                194    0.567  78.712   0.513   0.021   0.279   0.699
E F  4OZGE                195    0.311  62.357  -0.686   0.021   0.279   0.699
E F  4OZGE                196    0.294  59.006  -1.263   0.004   0.197   0.799
E P  4OZGE                197    0.579  82.174   0.009   0.004   0.085   0.910
E S  4OZGE                198    0.393  46.083   0.128   0.018   0.047   0.935
E P  4OZGE                199    0.632  89.638  -1.229   0.018   0.047   0.935
E E  4OZGE                200    0.655 114.428   0.266   0.018   0.088   0.893
B S  4OZGE                201    0.173  20.311  -2.122   0.018   0.047   0.935
E S  4OZGE                202    0.774  90.666  -1.383   0.003   0.003   0.994

Explain the output.  Go back.
```

---
